# Understanding Transcriptomic and Serological Differences between Forced Molting and Natural Molting in Laying Hens

**DOI:** 10.3390/genes13010089

**Published:** 2021-12-29

**Authors:** Tongyu Zhang, Zhonghua Ning, Yu Chen, Junhui Wen, Yaxiong Jia, Liang Wang, Xueze Lv, Weifang Yang, Changqing Qu, Haiying Li, Huie Wang, Lujiang Qu

**Affiliations:** 1 State Key Laboratory of Animal Nutrition, Department of Animal Genetics and Breeding, National Engineering Laboratory for Animal Breeding, College of Animal Science and Technology, China Agricultural University, Beijing 100193, China; zhangty0611@126.com (T.Z.); ningzhh@cau.edu.cn (Z.N.); wjh8545@cau.edu.cn (J.W.); 2 Beijing Animal Husbandry and Veterinary Station, Beijing 100107, China; chenyu.cncn@163.com (Y.C.); wangliangcau@139.com (L.W.); lvxueze0310@163.com (X.L.); carspstp@126.com (W.Y.); 3 Institute of Animal Science, Chinese Academy of Agricultural Sciences, Beijing 100193, China; jiayaxiong@caas.cn; 4 Engineering Technology Research Center of Anti-Aging Chinese Herbal Medicine of Anhui Province, Fuyang Normal University, Fuyang 236037, China; qucq518@163.com; 5 College of Animal Science, Xinjiang Agricultural University, Urumqi 830052, China; lhy-3@163.com; 6 College of Animal Science, Tarim University, Alar 843300, China; whedky@126.com

**Keywords:** forced molting, natural molting, serological indices, transcriptomic analysis, chickens

## Abstract

Molting is natural adaptation to climate change in all birds, including chickens. Forced molting (FM) can rejuvenate and reactivate the reproductive potential of aged hens, but the effect of natural molting (NM) on older chickens is not clear. To explore why FM has a dramatically different effect on chickens compared with NM, the transcriptome analyses of the hypothalamus and ovary in forced molted and natural molted hens at two periods with feathers fallen and regrown were performed. Additionally, each experimental chicken was tested for serological indices. The results of serological indices showed that growth hormone, thyroid stimulating hormone, and thyroxine levels were significantly higher (*p* < 0.05) in forced molted hens than in natural molted hens, and calcitonin concentrations were lower in the forced molted than in the natural molted hens. Furthermore, the transcriptomic analysis revealed a large number of genes related to disease resistance and anti-aging in the two different FM and NM periods. These regulatory genes and serological indices promote reproductive function during FM. This study systematically revealed the transcriptomic and serological differences between FM and NM, which could broaden our understanding of aging, rejuvenation, egg production, and welfare issues related to FM in chickens.

## 1. Introduction

Feathers are an integral part of the life of all birds and provide protection, insulation, and strength during flight and courtship. Natural molting (NM) occurs in birds as an adaptive mechanism to climate change [1]. For commercial laying hens, the function of feathers is mainly limited to coping with heat stress in special environments and avoiding close scratches during feeding. Molting in laying hens is a natural and orderly process, similar to that in other birds. Under the direct or indirect action of hormones such as thyroxine, estrogen, and testosterone, wing feathers are the first to molt, followed by body feathers [2]. Among them, feathers from the abdomen and back of chickens are shed the most.

As with all birds, most chickens would molt each fall, although the time of molting varies slightly among different bird species under different conditions [3]. Generally, it would take 14–16 weeks to complete NM, whereas forced molting (FM) can be completed in 4 weeks for chickens without being affected by seasons. In addition, egg-laying rate (ELR) and egg quality were improved after FM, whereas the ELR decreased during NM due to aging. Therefore, compared with NM, FM does not only achieve centralized and unified molting in a short time to facilitate production management, but also improves the ELR, egg quality, and even increases the utilization life of aged hens. In addition, FM effectively improves intestinal health [4] and strengthens bone calcium metabolism to avoid a variety of rickets in aged hens [5]. Therefore, FM has been increasingly applied in poultry production to improve longevity and the ELR in aged hens.

FM is the artificial application of some external pressure to layers to make the hens stop laying eggs, lose weight (<30%), and lose feathers. The hens recover their strength by regaining their previous weight and growing new feathers until the ELR gradually returns to the second peak of egg production [6]. The common methods of FM are fasting, hormonal, chemical, and combined (fasting-chemical) methods. Owing to its simple operation, low cost, and obvious effect, fasting is the most popular method of FM. 

Older chickens over 500 days old are more likely to produce broken and soft-shell eggs due to the disorder of vitamin D3 metabolism, which affects the absorption of blood calcium [7]. With an increase in feeding time, fat deposition in the body of the old hens increases significantly, and fat deposition around the uterine gland that secrets eggshell also increases, which further affects the formation of eggshell and reduces the quality of eggshell. FM can improve egg production and quality for many reasons. First, FM by fasting can consume excess fat in the body of laying hens causing a decrease in weight. When the fat deposited in the uterine glands is consumed, the function of eggshell secretion can be restored [8]. In addition, FM also leads to the enhancement of blood calcium metabolism, increases the intestinal calcium-binding protein concentration, and promotes the absorption of calcium in the intestine, thus improving the quality of eggshells [9]. Cell apoptosis, autophagy, and aging in the shell gland of the oviduct during starvation or cell remodeling and proliferation after the resumption of feed supply may also affect eggshell strength [10,11].

The hypothalamus can regulate the variation in the ELR in different types of laying hens [12]. The ovary is not only an important reproductive organ, but also a sensitive organ that responds to aging [13]. In humans, age-associated decreases in follicle number and oocyte quality decrease fecundity due to oxidative stress [14]. The hypothalamic-pituitary-gonadal axis has an obvious effect on prolonged laying time and improves the fecundity of laying hens [15]. Therefore, the gene expression level of hypothalamus and ovary can be used as an important basis for the response of FM to external environmental changes, such as aging or fasting.

With the development of next-generation sequencing, transcriptome techniques have been widely used to reveal the genetic mechanisms of reproduction [16], aging [17], development [18], and disease resistance traits [19] in chickens. However, to date, it is not known how FM and NM vary at the hormonal and genetic levels. In the present study, we focused on the hormonal and genetic variations in FM over NM using RNA-seq. Therefore, the main goal of this study was to identify differentially expressed genes (DEGs) between heavy shedding and the regrowth of feathers during FM over NM. We characterized and quantified mRNAs that are expressed in the hypothalamus and ovary during the different periods of FM and NM in domestic chickens. This study reveals the differences in FM from the perspective of hormones and genetics to broaden our understanding of aging, rejuvenation, egg production, and welfare issues related to FM in chickens.

## 2. Materials and Methods

### 2.1. Chickens 

All experiments were approved by the Committee for Animal Care and Use of China Agricultural University (approval ID: XXCB-20090209). The experimental procedures were performed according to the Guidelines for Experimental Animals established by the Ministry of Science and Technology (Beijing, China).

### 2.2. Animal Experimental Design 

A total of 44,079 Jingfen No. 6 laying hens were selected for FM at the laying farm of the Hubei Shendan Company (Anlu, China). All chickens were housed in battery cages in an enclosed house. When the chickens were 456 days old, the ELR decreased to 0.774, and FM was induced by fasting for 12 days with initial 2 days without water until egg production completely ceased. At the same time, chickens were exposed to 8 h of light and 16 h of darkness (8 L:16 D) during FM. The average room temperature was 18.4 °C and the humidity was 45.9%. The fasting termination time was determined when the average percentage of body weight loss in all hens was approximately 30%. At this time (FM_1), three 469 days old chickens with similar body weights and health conditions were selected for slaughter. Serological indices were detected in the blood of the three chickens, and hypothalamic and ovarian tissue samples were collected. Feed and water were then gradually supplied to allow the layers to regain weight and egg production, and new feathers regrew during this period. By 527 days (FM_2), the ELR returned to its second peak (87.3%). Three healthy hens with uniform body weight were selected from the chicken group and slaughtered. Blood samples were collected for serological index determination, and hypothalamic and ovarian tissue samples were collected for RNA-seq (Table 1).

There were also 2500 natural molted chickens that were fed in a breeding base (Zhuozhou, China). All chickens were housed in the same cage and fed the same nutritious diet. When the chickens were 572 days old (NM_1), their feather loss was very serious, and the ELR was 0.417. Then, three healthy hens with uniform body weight were selected from the chicken group and slaughtered to collect blood samples for serological index determination, and tissue samples from the hypothalamus and ovaries were collected for transcriptome sequencing. When the chickens were 721 days old (NM_2), they regrew new feathers, but with lower ELR (0.251), and a second sample collection was performed for NM. Three healthy hens with uniform body weight were selected from the chicken group and slaughtered to collect blood samples for serological index determination, and hypothalamic and ovarian tissue samples were collected for RNA-seq (Table 1).

### 2.3. Serological Indices

All blood samples from FM and NM experimental chickens were incubated overnight at 4 °C. The serum was separated using centrifugation at 20,000× *g* for 5 min in anticoagulant tubes. Growth hormone (GH), thyroid stimulating hormone (TSH), calcitonin (CT), and thyroxine (T4) levels were measured at the Bioygene Biological Technology Co. Ltd. (Wuhan, China) using ELISA. The student’s *t*-test was used to analyze the differences in the serological indices between the two periods. 

### 2.4. Sample Collection

The chicken skull was opened to collect 100–200 mg of the hypothalamic tissue, and the abdomen of the chicken was dissected to collect approximately 200 mg of the ovarian tissue. All tissue samples were washed in 0.9% normal saline, placed in RNA-free storage tubes, frozen in liquid nitrogen, and stored at −80 °C.

### 2.5. RNA Isolation, Library Construction, and Sequencing

Total RNA from the hypothalamic and ovarian tissues of 12 chickens was extracted using TRIzol reagent (Invitrogen, Carlsbad, CA, USA). RNA concentration was measured using a NanoDrop ND2000 spectrophotometer (NanoDrop Products, Wilmington, NC, USA), according to the manufacturer’s instructions. An Agilent 2100 Bioanalyzer (Agilent Technologies, CA, USA) was used to detect the integrity and concentration of the extracted RNA using agarose gel electrophoresis.

Next, high-quality RNA samples (concentration > 50 ng/μL, OD260/280 = 1.8–2.2, OD260/230 = 1.8–2.2, RIN > 8, 28S:18S ≥ 0.5) were used to construct sequencing libraries. Poly(A) mRNA was isolated from the total RNA samples with oligo (dT) magnetic beads (Invitrogen), and the RNA was broken to a fragment of approximately 300 bp in length using ion breaking (Qiagen GmbH, Hilden, Germany). The first cDNA strand was synthesized from mRNA using 6-base random primers and reverse transcriptase and the second cDNA strand was synthesized using the first cDNA strand as a template. The cDNA was synthesized by reverse transcription PCR using Prime Script™ RT Reagent kit (Takara, Dalian, China). Then, the resultant cDNA was preserved at −20 °C. PCR amplification was used T7 and SP6 primers under the following conditions: 95 °C for 5 min followed by 35 cycles at 94 °C for 30 s, 55 °C for 30 s, 72 °C for 2 min, and a final cycle at 72 °C for 5 min. PCR products were then analyzed on 2% agarose gels and sequenced. Libraries were selected for cDNA target fragments of 450 bp, followed by PCR amplification. A directional cDNA library of the samples of the chicken was constructed using the pSPORT-1 SuperScript Plasmid Cloning System from Gibco/BRL, and the library was electroporated into ElectroMax DH12S cells. Random clones were grown in 1.5-mL LB medium overnight in 12 × 75 mm culture tubes. Then, high-throughput sequencing was performed using cycleSeq-farOUTTM polymerase (Display Systems Biotech, Vista, CA, USA). The purified cDNA libraries were prepared using TruSeqTM DNA XXmple Prep Kit-Set A (Illumina, San Diego, CA, USA) and amplified using TruSeq PE Cluster Kit (Illumina Inc., San Diego, CA, USA). Ultimately, a total of 24 libraries were sequenced with 150 bp paired-end reads using the Illumina HiSeqTM2500 (Illumina Inc.) at Shanghai Personal Biotechnology Co. Ltd. (Shanghai, China).

### 2.6. Alignment with Reference Genome and DEG Analysis

Raw FASTQ data were first checked for high quality using fastp software with default parameters to remove joints, blank reads, and low-quality sequences (sequences with N ratio of >10% and Q-value of < 20%). High-quality clean reads were obtained for subsequent analysis. Then, HISAT2-2.2.0 software was used to map clean reads to the reference genome Gallus_gallus-5.0 (https://www.ncbi.nlm.nih.gov/assembly/GCF_000002315.6, accessed on 27 March 2021). Gene expression quantification was performed using the normalized numbers of the fragments per kilobase of transcript per million mapped reads (FPKM) method. We then performed RNA-seq analysis of hypothalamus and ovarian tissues of force molted and normal molted hens using the DESeq R package. DEGs were finally selected using a cutoff at a false discovery rate (FDR) of <0.05 and |log2FoldChange| ≥ 1.

### 2.7. Functional Annotation of DEGs

GO analysis is a commonly used approach for defining genes and their RNA or protein products by identifying the unique biological properties of high-throughput transcriptome or genome data. Based on the GO terms of transcripts, these DEGs were assigned into three main functional categories: biological process (BP), cellular component (CC), and molecular function (MF). KEGG is a collection of databases dealing with genomes, diseases, biological pathways, and chemical materials. DAVID (https://david.ncifcrf.gov/tools.jsp, accessed on 16 May 2021), an online bioinformatics tool, was designed to identify a large number of DEGs or protein functions for GO and KEGG pathway analyses. 

## 3. Results

### 3.1. Comparison of Changes in Feather Coverage between Two Molting Modes

**Figure 1:**
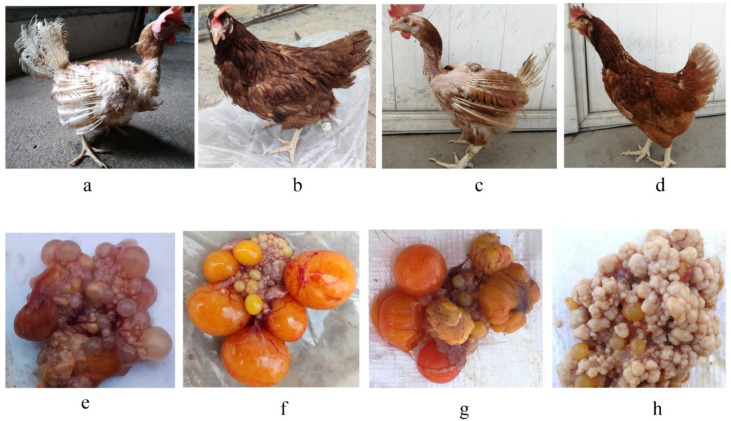
Feather coverage and corresponding ovarian tissue in forced molted and natural chickens for two different periods. A chicken that shed many feathers during the FM_1 period (**a**) and its corresponding ovarian tissue (**e**); A chicken with regrown feathers during the FM_2 period (**b**) and its corresponding ovarian tissue (**f**); A chicken that shed many feathers during the NM_1 period (**c**) and its corresponding ovarian tissue (**g**); A chicken with regrown feathers during the NM_2 period (**d**) and its corresponding ovarian tissue (**h**).

There was a little difference between the two molting modes in the terms of feather coverage, but the appearance of the ovaries varied greatly (Figure 1). There were no fertilized eggs on the ovaries of hens in the FM_1 period (Figure 1e) as the ovaries were in a state of atrophy, whereas many brightly colored fertilized eggs were attached to the ovaries in the FM_2 period (Figure 1f). Interestingly, the overall color of the ovarian tissue was darker both in the NM_1 and NM_2 periods (Figure 1). In the NM_1 period, a few fertilized eggs were attached to the ovaries of the hens (Figure 1g), but there were almost no fertilized eggs attached to the ovary tissue, and ovarian cysts appeared in the NM_2 period (Figure 1h).

### 3.2. Detection of Serological Indices between FM and NM 

The serological indices of the two periods of FM and NM were examined, and a *t*-test was conducted to analyze the four groups of data (Table 2). From the results, FM and NM had a great effect on serological indices, but there was little change in the serological indices in the different periods with the same molting pattern. GH, TSH, and T4 were significantly higher in the force molted than in the normal molted hens (*p* > 0.05); however, there was no significant difference in the content of these three hormones in the different molting periods of the two molting modes (*p* < 0.05). In contrast, CT was significantly lower in the FM group than in the NM group (*p* > 0.05). There were no significant hormonal changes for FM_1 and FM_2, but changes in CT during two periods of NM were significant (Table 2).

### 3.3. RNA-Seq Analysis for Identifying DEGs among Eight Groups

**Figure 2:**
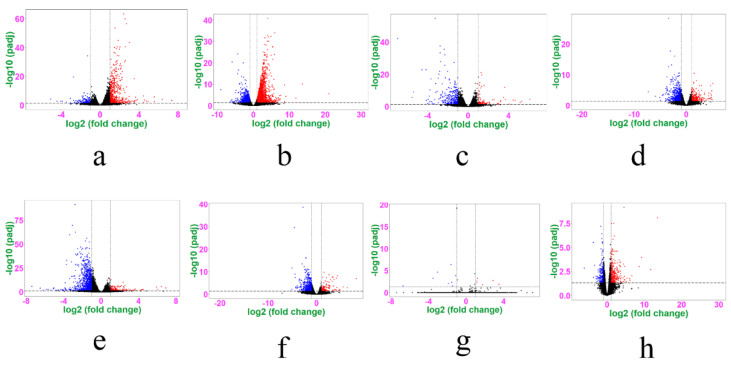
Volcano plot of eight groups of DEGs (hypothalamus and ovary from FM_1-vs-NM_1, FM_2-vs-NM_2, FM_1-vs- FM_2, and NM_1-vs- NM_2 groups). The hypothalamus (**a**) and ovary (**b**) in the FM_1 -vs- NM_1, hypothalamus (**c**), and ovary (**d**) in the FM_2 -vs- NM_2; hypothalamus (**e**) and ovary (f) in the FM_1 -vs- FM_2, and hypothalamus (**g**) and ovary (**h**) in the NM_1 -vs- NM_2 groups. The abscissa represents the fold change in gene expression in the hypothalamic and ovarian samples of forced molted hens compared with natural molted hens. The ordinate represents the significance of the difference in the number of genes expressed. The red dot indicates the significantly upregulated genes (Fold Change > 2, FDR < 0.05), and the blue dot indicates the significantly downregulated genes (Fold Change > 2, FDR < 0.05).

The clean reads of the 24 samples were all high quality, with Q20 > 97% and Q30 > 92%, and the GC content was approximately 50% (Table S1). A total of 24,104 detectable genes were expressed in both the hypothalamus and ovaries. We subsequently performed four differential expression analyses of the hypothalamus and ovaries in the same period of the two different molting modes, and then another four groups of RNA-seq analyses were conducted for the hypothalamus and ovaries during different periods of the same molting mode, ultimately obtaining a total of eight groups of DEGs. Volcano maps were obtained using transcriptome analysis of the eight sets of data separately (Figure 2). The FPKM hierarchical cluster analysis showed that the expression of DEGs in the eight groups was accurately distinguished and the data were repeatable and credible (Supplementary Figure S1).

**Figure 3:**
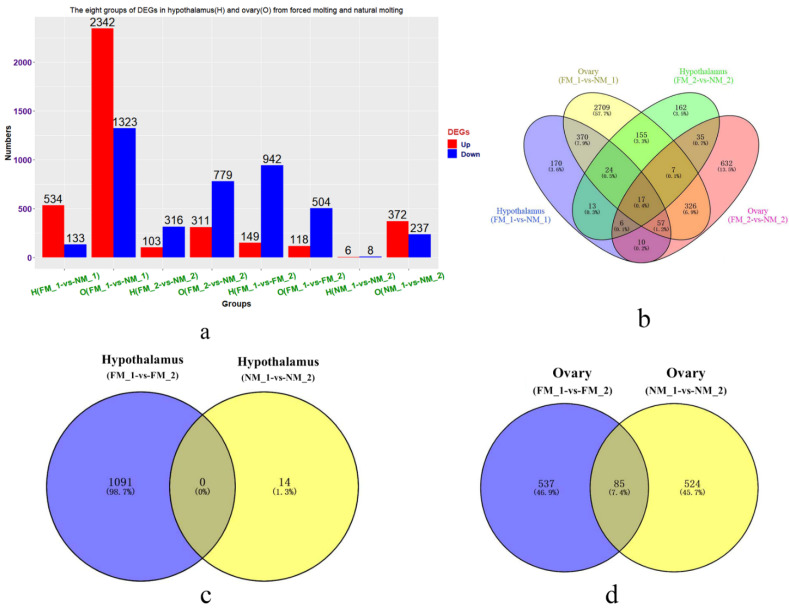
Description of eight groups of DEGs (hypothalamus and ovary from FM_1-vs-NM_1, FM_2-vs-NM_2, FM_1-vs- FM_2, and NM_1-vs- NM_2 groups). The numbers of DEGs in hypothalamus and ovary of forced molted and natural molted hens (**a**), The red indicates the significantly upregulated genes, and the blue indicates the significantly downregulated genes. The Venn diagram of four groups of DEGs from the same molting periods for the two different molting patterns (**b**). The Venn diagram of DEGs in the hypothalamus (**c**) and ovary (**d**) in the FM_1-vs- FM_2 and NM_1-vs- NM_2 groups.

Overall, the number of DEGs between FM and NM significantly varied in different periods and tissues, and there were several common DEGs between the different groups (Figure 3). Among them, the ovaries in the FM_1-vs-NM_1 group had the most DEGs (2342 upregulated DEGs and 1323 downregulated DEGs), whereas the hypothalamus in the NM_1-vs-NM_2 group had the least number of DEGs (six upregulated DEGs and eight downregulated DEGs) (Figure 3a). In addition, the Venn diagram of the four groups (hypothalamus and ovaries in the FM_1-vs-NM_1 and FM_2-vs-NM_2 groups) of DEGs showed that there were intersections among them, with 17 common DEGs (Figure 3b). There were no common DEGs in the hypothalamus between the two different molting patterns (Figure 3c), whereas there were 85 common DEGs in the ovaries between the two different molting patterns (Figure 3d).

### 3.4. DEGs in Hypothalamus in FM_1-vs-NM_1 Group

**Figure 4:**
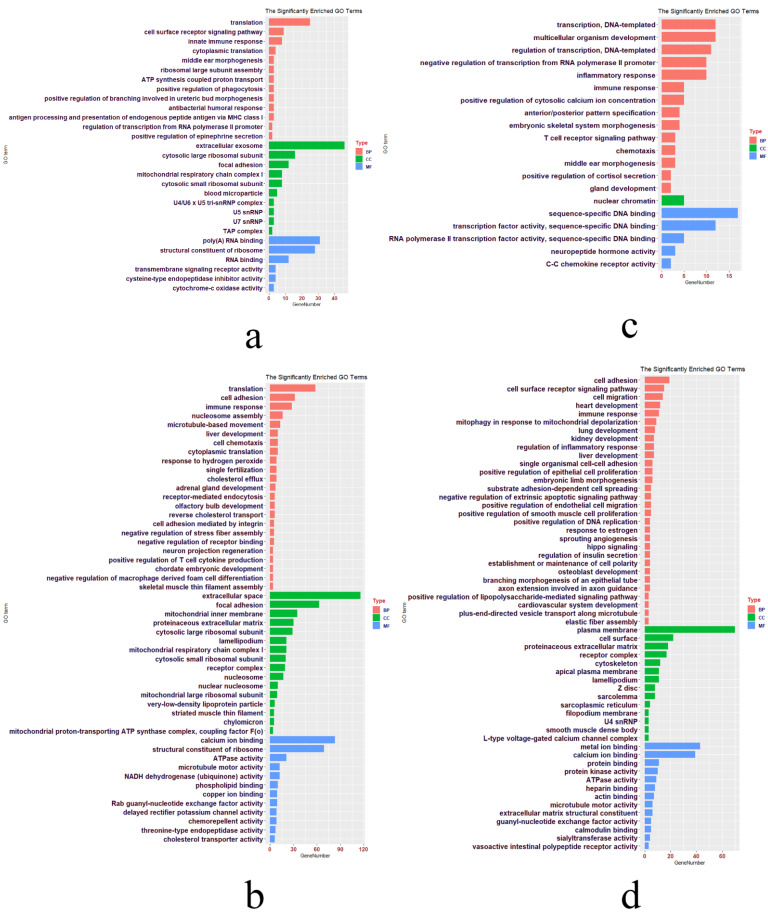
GO terms for four groups of DEGs (hypothalamus and ovary in FM_1-vs-NM_1 and FM_2-vs-NM_2). BP refers to biological process, CC refers to cellular component, and MF refers to molecular function. The GO terms for the hypothalamus (**a**) and ovary (**b**) in the FM_1 -vs- NM_1 group. The GO terms for the hypothalamus (**c**) and ovary (**d**) in the FM_2 -vs- NM_2 group.

**Figure 5:**
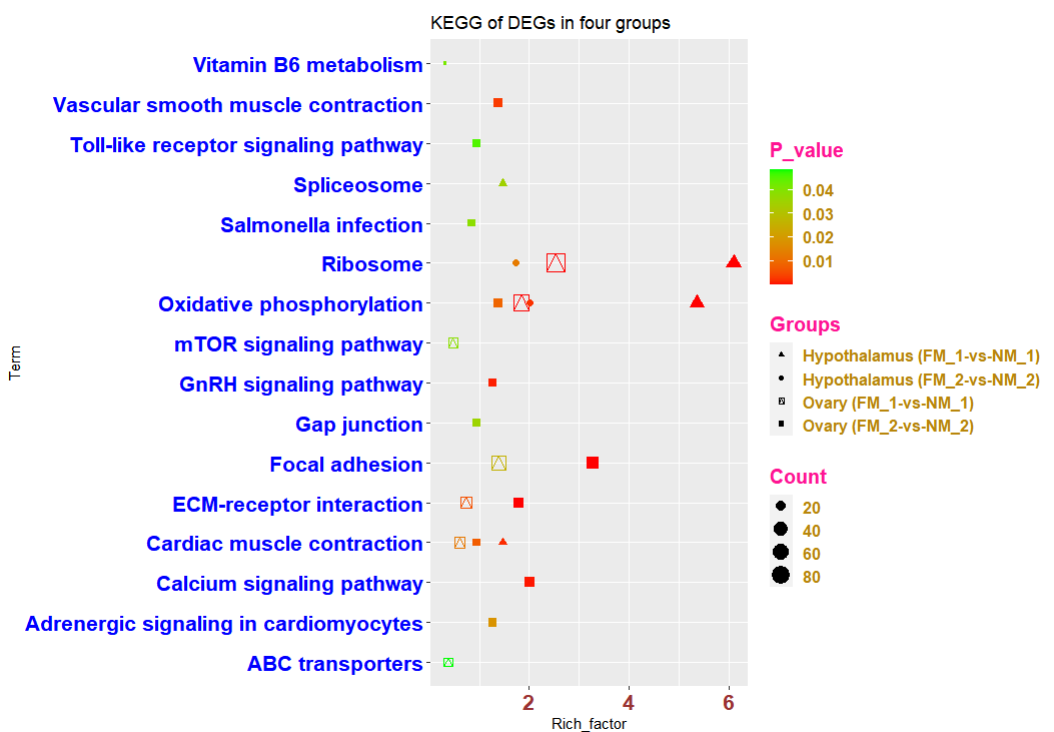
KEGG pathways for four groups of DEGs (hypothalamus and ovary in FM_1-vs-NM_1 and FM_2-vs-NM_2) in the same periods for different molting patterns.

A total of 667 DEGs in the hypothalamus in the FM_1-vs-NM_1 group were used to perform GO term and KEGG pathway analyses (Additional file S1). Finally, 29 significant GO terms (13 BP, 6 MF, and 10 CC) and four KEGG pathways were enriched (Additional file S2, Figure 4a and Figure 5). GO terms are mainly enriched in immune-related terms, such as innate immune response, antigen processing, and the presentation of endogenous peptide antigens via MHC class I, antibacterial humoral response, and the positive regulation of phagocytosis. There are also pathways related to protein synthesis and metabolism, such as translation, cytoplasmic translation, ATP synthesis coupled proton transport, positive regulation of epinephrine secretion, and cytochrome-c oxidase activity. Furthermore, a number of GO terms related to ribosomal synthesis were also enriched, such as cytosolic large ribosomal subunit, cytosolic small ribosomal subunit, structural constituent of ribosome, ribosomal large subunit assembly, U7 snRNP, U5 snRNP, and U4/U6 × U5 tri-snRNP complex. The four significant KEGG pathways were ribosome, oxidative phosphorylation, cardiac muscle contraction, and spliceosome. 

### 3.5. DEGs in Ovaries in FM_1-vs-NM_1 Group

The FM by fasting by can cause ovarian shrinkage and a lower ELR in chickens, however, their ELR will gradually increase with subsequent water supply and feeding. In contrast, the egg production rate of NM decreased all the time. There were 3665 DEGs in the ovaries in the FM_1-vs-NM_1 group (Additional file S3). A total of 51 GO terms and seven KEGG pathways were significantly enriched (*p* < 0.05) using 3665 DEGs (Additional file S4, Figure 4b and Figure 5). GO terms were enriched in immune-related terms, such as immune response, positive regulation of T cell cytokine production, negative regulation of macrophage-derived foam cell differentiation, and receptor-mediated endocytosis. In addition, many developmental pathways were enriched, such as olfactory bulb development, chordate embryonic development, liver development, adrenal gland development, and cell adhesion. There are also many GO terms related to protein synthesis and energy metabolism, such as translation, mitochondrial inner membrane, mitochondrial large ribosomal subunit, mitochondrial proton-transporting ATP synthase complex, coupling factor F(o), structural constituents of ribosome, and dehydrogenase (ubiquinone) activity. The seven significant KEGG pathways were ribosome, oxidative phosphorylation, ECM-receptor interaction, cardiac muscle contraction, focal adhesion, mTOR signaling pathway, and ABC transporters.

### 3.6. DEGs in Hypothalamus in FM_2-vs-NM_2 Group

There were 419 DEGs of hypothalamus in FM_2-vs-NM_2 group (Additional file S5) performed GO terms and KEGG pathways. Finally, a total of 20 GO terms (14 BP, 1 CC, and 5 MF) and two KEGG pathways were significantly enriched (*p* < 0.05) based on these 419 DEGs (Additional file S6, Figure 4c and Figure 5). GO terms were mainly enriched in the development of organs and tissues, such as multicellular organism development, gland development, and embryonic skeletal system morphogenesis. In addition, some immune-related GO terms (inflammatory response, T cell receptor signaling pathway, and immune response) were also enriched. Other enriched GO terms included transcription factor activity, sequence-specific DNA binding, RNA polymerase II transcription factor activity, sequence-specific DNA binding, RNA polymerase II transcription factor activity, sequence-specific DNA binding, transcription, the regulation of transcription, DNA template formation, and sequence-specific DNA binding. Oxidative phosphorylation and ribosomes were the only two enriched KEGG pathways.

### 3.7. DEGs in Ovaries in FM_2-vs-NM_2 Group

A total of 1090 DEGs in the ovaries in FM_2-vs-NM_2 group (Additional file S7) were significantly enriched (*p* < 0.05) in 57 GO terms and 12 KEGG pathways (Additional file S8, Figure 4d and Figure 5). GO terms were mainly enriched in the development of cells and organs, such as lung development, liver development, embryonic limb morphogenesis, kidney development, osteoblast development, and cardiovascular system development. In addition, two GO terms (branching morphogenesis of an epithelial tube and positive regulation of endothelial cell migration) of skin cell proliferation were also enriched. Several GO terms related to cell activity were also enriched, such as cell adhesion, cell surface receptor signaling pathway, cell migration, positive regulation of smooth muscle cell proliferation, single organismal cell-cell adhesion, extracellular matrix structural constituent, cytoskeleton, and cell surface. Furthermore, calcium signaling pathway, calcium ion binding, calmodulin binding, and L-type voltage-gated calcium channel complex involved in calcium metabolism and synthesis were also enriched. Similar to the annotated terms for the other groups, DEGs were also enriched in some GO terms related to immunity (immune response), mitochondrial metabolic activity, DNA transcription, and translation.

### 3.8. DEGs in Hypothalamus and Ovaries in FM_1-vs-FM_2 and NM_1-vs-NM_2 Groups

**Figure 6:**
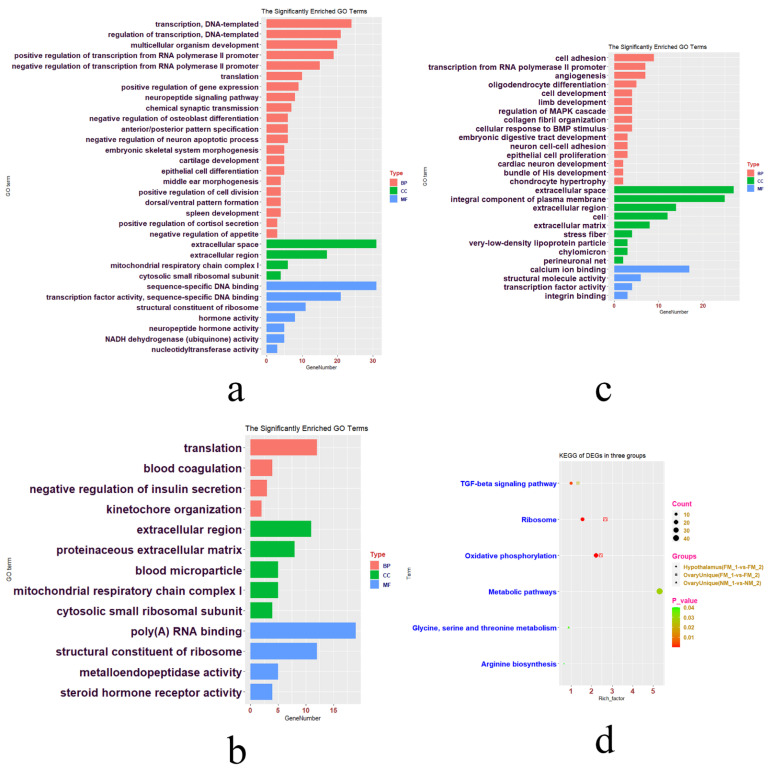
GO terms and KEGG pathways for three groups of DEGs in same periods for different molting patterns. The significant GO terms for the hypothalamus in the FM_1-vs-FM_2 group (**a**). The significant GO terms for unique 537 ovarian DEGs in the FM_1-vs-FM_2 group (**b**) and unique 524 ovarian DEGs in the NM_1-vs-NM_2 group (**c**). The significant KEGG pathways of the above three group of DEGs.

Gene expression in the hypothalamus and ovaries varied greatly between the FM_1-vs-FM_2 and NM_1-vs-NM_2 groups (Additional file S9, Figure 3c,d). A total of 1091 hypothalamic DEGs in the FM_1-vs-FM_2 group were enriched in 21 BP (multicellular organism development, neuropeptide signaling pathway, and negative regulation of osteoblast differentiation), four CC (extracellular space, mitochondrial respiratory chain complex I, extracellular region, and cytosolic small ribosomal subunit), seven MF (sequence-specific DNA binding, hormone activity, and neuropeptide hormone activity), and four KEGG pathways (oxidative phosphorylation, ribosome, TGF-beta signaling pathway, and metabolic pathways) (Additional file S10, Figure 6a). However, the number of 14 DEGs in the hypothalamus (NM_1-vs-NM_2) was too small to be analyzed by DAVID, and only four genes function were effective. 

There were 537 unique ovarian DEGs in the FM_1-vs-FM_2 group and 524 unique ovarian DEGs in the NM_1-vs-NM_2 group (Additional file S11, Figure 3d). The 537 unique ovarian DEGs in the FM_1-vs-FM_2 group were enriched in four BP (translation, blood coagulation, negative regulation of insulin secretion, and kinetochore organization), five CC (mitochondrial respiratory chain complex I, blood microparticle, proteinaceous extracellular matrix, cytosolic small ribosomal subunit, and extracellular region), four MF (structural constituent of ribosome, steroid hormone receptor activity, poly(A) RNA binding, and metalloendopeptidase activity), and three KEGG pathways (ribosome, oxidative phosphorylation, and TGF-beta signaling pathway) (Additional file S12, Figure 6b,d). In addition, 524 unique ovarian DEGs in other groups were enriched in 15 BP (oligodendrocyte differentiation, cellular response to BMP stimulus, and cell adhesion), nine CC (cell, extracellular space, and integral component of plasma membrane), four MF (calcium ion binding, transcription factor activity, RNA polymerase II distal enhancer sequence-specific binding, structural molecule activity, and integrin binding), and two KEGG pathways (arginine biosynthesis, glycine, serine, and threonine metabolism) (Additional file S12, Figure 6c,d). 

Subsequently, GO Terms and KEGG pathways of all the above groups were classified and summarized according to their specific functions, and it was found that these DEGs were mainly enriched in GO Terms and KEGG pathways related to immunity, development, aging, energy metabolism, health, and other functions (Table S2). Finally, 23 DEGs were screened from the above GO Terms and KEGG pathways with representative function in each group (Table S3), there are also four hypothalamic DEGs (NM_1-vs-NM_2) listed separately in this table. The level of expression of these 27 genes helps explain their function in each group.

## 4. Discussion

In our study, the age of FM chickens was 469 and 527 days, with the study lasting for 58 days, while during the two NM periods, the age of hens were 572 days and 721 days, lasting for 149 days. FM was similar to NM in the pattern of changed feather coverage, but the time of molting and ELR were greatly different, resulting from remarkable differences in intrinsic hormone and genetic regulation. Anatomy experiments also showed a wide variation in the appearance of the ovaries.

After FM, the ovarian tissue showed obvious atrophy without attached fertilized eggs. However, when the chickens returned to the second peak of egg production, ovarian tissues regained development, and they were also attached with brightly colored fertilized eggs. Taken together, FM can effectively restore the reproductive function of the ovaries in laying hens. However, the NM situation is not optimistic. During the molting process, the ovarian tissue was relatively normal, with some fertilized eggs attached to it. Although the feathers regrew after molting, the ovarian tissue was dull and had cysts. There is no melanocytic component in the normal ovarian tissue [20], and these symptoms are caused by the deposition of melanoma [21] and ovarian cysts [22] caused by senility. Therefore, it was once again proven that layers became weaker during the NM process, but FM can improve the health of laying hens [6].

Serological tests were performed on 12 forced molted and normal molted chickens to understand the genetic and serological differences between FM and NM in hens. Although having a small sample size (n = 3), each experimental chicken was carefully screened to ensure the same weight and health between individuals. Furthermore, the mean values of any two sets of data with significant differences showed at least a two-fold difference. Therefore, our serological test data have value as a reference. Overall, the different molting methods had a remarkable effect on serological indices. During the fasting period of laying hens, the secretion of GH increases and promotes the formation and development of follicles by regulating cell proliferation and apoptosis [23]. In addition, the concentrations of T4 and TSH in the natural molted chickens were lower than those in forced molted chickens. TSH can promote the secretion of T4 by the thyroid, thus strengthening animal metabolism and ontogenesis [24,25]. Taken together, compared with NM, FM can stimulate the life potential of laying hens through the action of hormones, resulting in a more vigorous metabolism and stronger reproduction. CT is secreted through thyroid gland and stimulates osteoblast growth to prevent calcium loss [26]. The difference of CT between NM_1 period and NM_2 period might be explained by the difference in age of the two groups. While the CT levels in FM groups is multifactorial, starvation causes lower blood calcium concentration, lower activity of gastrointestinal hormones, etc., which might explain the lower concentration of calcitonin activity at FM_1 and similar levels (because of the age) at the FM_2 period. Above all, serological indices can contribute to reveal the inherent differences of FM over NM to some extent.

Further transcriptome analysis showed that there were more DEGs in the ovary than in the hypothalamus, indicating that the ovary was more sensitive than the hypothalamus to changes in the external environment during the FM process. However, the number of DEGs in the hypothalamus and ovary during the FM process was higher than that during the NM process. The results also proved that FM is a process of drastic change in genetic expression profile for hens, whereas it changes unremarkably during NM periods. This may be caused by hormone metabolism disorders in chickens under starvation stress of FM, leading to apoptosis in ovarian granules and the inhibition of follicle formation, eventually resulting in ovarian atrophy [23]. However, the ovaries of laying hens resumed their reproductive potential when feed and water were restored. 

Firstly, we focused on the genetic change mechanism of the two molting modes in the same period, and four groups of DEGs were ultimately obtained. On the whole, four groups of DEGs between FM and NM were all annotated into immune-related pathways, showing that the decrease in physical fitness requires the activation of the immune system to maintain a healthy balance in the process of molting for the aged chickens. The DEGs of hypothalamus in the group of FM_1-vs-NM_1 were enriched to four immune-related GO terms (antigen processing and presentation of endogenous peptide antigen via MHC class I, antibacterial humoral response, innate immune response, and positive regulation of phagocytosis). MX1 and PTX3 were two higher expressed representative genes in GO term of innate immune response. These two genes were all higher expressed in FM_1, compared to NM_1. MX1 is an antiviral gene and has been shown to have a role in avian flu resistance [27]. PTX3 can be used as a marker to detect the health of chickens, with the expression of PTX3 known to increase in chickens after viral and bacterial infection [28]. These results suggest that FM can activate the immune stress function of laying hens under starvation stress. In addition, the hypothalamus plays an important role in regulating energy metabolism during FM by fasting. Therefore, hypothalamic DEGs in the FM_1-vs-NM_1 group were enriched in GO terms (ATP synthesis coupled proton transport, positive regulation of epinephrine secretion, cytosolic large ribosomal subunit, cytosolic small ribosomal subunit, structural constituent of ribosome, ribosomal large subunit assembly, and cytochrome c oxidase activity) and KEGG pathways (Cardiac muscle contraction) related to energy metabolism. Ribosomes are important sites of protein synthesis. The mutations in the mitochondrial genes of cytochrome c oxidase (COX) are prone to cause nerve cells to undergo apoptosis, thereby inhibiting energy metabolism and ultimately affecting ontogenesis [29].

The ovarian DEGs in the group of FM_1-vs-NM_1 were annotated in four immune-related GO terms (immune response, negative regulation of macrophage derived foam cell differentiation, positive regulation of T cell cytokine production and receptor-mediated endocytosis). In addition, several DEGs were enriched in GO terms related to cell development (olfactory bulb development, chordate embryonic development, liver development, and adrenal gland development) or aging (cell adhesion, NADH dehydrogenase (ubiquinone) activity, ABC transporters, mTOR signaling pathway, Focal adhesion, and Ribosome). SMAD3 can promote ovarian follicle development, and mutation of this gene can cause ovarian cancer [30]. SMAD3 was more highly expressed in NM_1 than FM_1. In fact, the atrophy and stunted development of ovary occurred in stage FM_1, while the ovaries develop normally in stage NM_1. CCL5 can promote the apoptosis of ovarian granulosa cells, damaging their development and maturation, causing ovarian aging [31]. CCL5 was more highly expressed in stage FM_1 to cause ovarian autophagy, aging and atrophy. In addition, four ovarian DEGs (ABCA5, ADIPOQ, ITGAV, and PPARG) in the FM_1-vs-NM_1 group are associated with negative regulation of macrophage-derived foam cell differentiation and are all involved in ovarian disease. ITGAV [32] and ABCA5 [33] have been reported to be associated with ovarian cancer and ADIPOQ [34] and PPARG [35] are associated with polycystic ovary syndrome. Among them, ADIPOQ was higher expressed in NM_1 while the other three genes were higher expressed in FM_1. Furthermore, compared with NM_1, FM_1 can activate the expression of three—ABCA5, ITGAV, and PPARG in the ovary and simultaneously inhibit the expression of ADIPOQ, thus causing the activation of negative regulation of macrophage derived foam cell differentiation. Additionally, under starvation stress, energy metabolism and autophagy of cells are enhanced to improve the adaptability of the stress environment by the regulation of hypothalamus. However, due to the atrophy of the ovary during the period of FM_1, several genes inhibiting ovarian development and accelerating cell aging were also annotated. Chordate embryonic development (FOXD3, WT1, BRCA1, and BRCA2) was enriched in DEGs in the ovaries. Demethylated FOXD3 decreases cell migration and proliferation abilities and increases cell apoptosis, even in ovarian cancer [36]. FOXD3 was more lowly expressed in FM_1 potentially contributing to accelerated cell aging, while the other three genes were higher expressed in FM_1. WT1 can regulate the apoptosis and proliferation of ovarian granulosa cells, and its mutations can cause abnormal ovarian development [37,38,39]. Upregulated BRCA1/BRCA2 levels can also cause ovarian cells to become cancerous [40].

The hypothalamic DEGs in the FM_2-vs-NM_2 group were mainly enriched in GO terms related to development of organs and tissues (multicellular organism development, gland development and embryonic skeletal system morphogenesis) in addition to Immune-related GO terms (inflammatory response, T cell receptor signaling pathway and immune response). The laying hens are supplied with feed to quickly recover the body function, accompanied by the redevelopment of organs and tissues after the FM, while the NM of hens is accompanied by aging and disease. HOXA3, HOXB3, HOXD4, HOXA4, and HOXB5 are involved in multicellular organism development and belong to the homeobox (HOX) family, which plays a crucial role in the early embryo development and cell differentiation [41]. All five genes of these genes were more highly expressed in FM_2 than NM_2. The ELR of our chickens at the FM_2 stage was also higher than in NM_2.

The ovarian DEGs in the FM_2-vs-NM_2 group were mainly enriched process connected with immune (cell adhesion, regulation of inflammatory response, single organismal cell-cell adhesion, substrate adhesion-dependent cell spreading, immune response and Salmonella infection), development (lung development, liver development, kidney development, osteoblast development, and cardiovascular system development), aging (Vitamin B6 metabolism, branching morphogenesis of an epithelial tube, and positive regulation of endothelial cell migration) and reproduction (calcium ion binding, Calcium signaling pathway, and GnRH signaling pathway). The calcium signaling pathway does not only regulate the animal endocrine system, development, proliferation, metabolism, and smooth muscle formation [42], but also promotes the enhancement of egg laying performance in aged chickens [43]. The GnRH signaling pathway is associated with egg production traits in chickens [12]. The ovaries redevelop in stage FM_2 and return to the second peak of egg production. The ovaries of chickens in stage NM_2 are gradually aging and diseased and the ELR also decreases significantly. MAP3K2 gene can inhibit cell proliferation and promote cell senescence and tumor occurrence [44]. We saw that MAP3K2 was over-expressed at stage NM_2. Therefore, MAP3K2 might play a vital role in ovarian aging and cyst formation. SLITRK5 was down-regulated at stage FM_2, and it has been reported that the inhibition of SLITRK5 expression can promote the development of ovarian granulosa cells [45].

To further reveal the genetic mechanism of FM and NM, we focused on the genetic changes in the same molting mode at two different periods. There were many more hypothalamic DEGs in FM_1-vs-FM_2 than in the NM_1-vs-NM_2 group. Most of the hypothalamic DEGs (FM_1-vs-FM_2) were mainly enriched in transcription, DNA-template formation, and multicellular organism development. The enriched KEGG pathways were associated with metabolic pathways, oxidative phosphorylation, and ribosomes. Therefore, FM can stimulate the metabolism of hens and play an anti-aging role. However, only four genes (CD69L, PDK4, HTR3A, and ZBTB32) were effective among the 14 hypothalamic DEGs (NM_1-vs-NM_2). PDK4, HTR3A, and ZBTB32 were more highly expressed in NM_2, meaning that their expression level increased gradually after NM. Upregulating PDK4 expression and improving mitochondrial activity can promote oocyte aging after ovulation [45,46]. The HTR3A gene plays an important role in regulating the genetic adaptation of birds to fear and reducing the side effects of stress responses [47]. However, the expression level of CD69L decreased gradually after NM. CD69L can activate the inflammatory response in pig skin endothelial cells [48], the decrease of its expression level can cause immunodeficiency in pigs. In short, the NM process is often accompanied by a decline in immune function as the body ages.

The ovary is sensitive to external changes, and there were several DEGs in the ovary found under both molting modes (there were 85 common DEGs). The unique DEGs of the ovary found in FM were mostly enriched in poly(A) RNA binding (EIF5B, SSB, etc.). EIF5B [49] and SSB [50] play crucial roles in the regulation of animal germ cell development, and these two genes were both more highly expressed in FM_2 than FM_1. The TGF-β signaling pathway is closely associated with embryo and bone development [51]. However, the unique DEGs of the ovary in NM were enriched in the KEGG pathways (Glycine, serine and threonine metabolism, and Arginine biosynthesis) related to health of hens. Arginine biosynthesis plays an important role in maintaining the health of older adults [52]. Feeding with glycine, serine, and threonine does not only promote the health of poultry [53], but also enhances growth performance and protein deposition in the carcass and viscera of pigs [54]. 

In this RNA-seq analysis, at two different periods of the same mode of molting, the hypothalamic DEGs and unique ovarian DEGs from the FM_1- vs-FM_2 comparison were all enriched in the KEGG pathways of oxidative phosphorylation (NDUFB6, NDUFS5, etc.). The ovary also showed an aging phenomenon in aged hens over 480 days old, mainly due to oxidative stress caused by accumulation of reactive oxygen species in the metabolic process [55]. Oxidative phosphorylation of mitochondria can cause mitochondrial dysfunction, resulting in cell apoptosis and aging [56,57]. NDUFB6 and NDUFS5, both over-expressed in FM_1, can lead to oxidative damage and apoptosis of myocardial cells [58]. Therefore, FM tends to be anti-aging for the ovary, and NM tends to maintain the health of aged chickens.

In conclusion, FM can rejuvenate aged hens and stimulate their reproductive potential, while the body of hens is continuously aging and even pathologically changes during NM, especially in the ovaries. Therefore, both FM and NM can result in re-molting, although the inherent genetic mechanisms are different. Both hormone levels and changes in gene expression are simultaneously involved in these complex physiological processes for FM and NM.

## Figures and Tables

**Table 1:** Chicken information for two periods of FM and NM.

Periods	Molting Type	Age (Days)	ELR	Description
FM_1	FM	469	0.002	Hens shed many feathers and ELR dropped to almost zero after fasting during FM
FM_2	FM	527	0.873	Hens grew new feathers and ELR returned to second peak of egg production after FM
NM_1	NM	572	0.417	Hens shed many feathers during NM
NM_2	NM	721	0.251	Hens grew new feathers after NM

FM, forced molting; NM, natural molting; ELR, egg-laying rate.

**Table 2:** Serological indices of FM and NM at different periods.

Serological Indices	FM_1	FM_2	NM_1	NM_2
GH (ng/mL)	12.77 ± 2.27 ^a^	16.98 ± 2.99 ^a^	5.16 ± 0.33 ^b^	5.30 ± 0.19 ^b^
TSH(µIU/mL)	11.89 ± 1.91 ^a^	13.16 ± 1.20 ^a^	3.95 ± 0.45 ^b^	4.47 ± 0.31 ^b^
CT(ng/L)	41.55 ± 6.51 ^a^	41.99 ± 8.36 ^a^	132.67 ± 3.60 ^b^	94.71 ± 2.41 ^c^
T4(ng/mL)	191.08 ± 18.39 ^a^	169.57 ± 31.80 ^a^	21.56 ± 0.94 ^b^	19.22 ± 1.46 ^b^

Note: Values in the same row with the same or no letter superscripts mean no significant difference (*p* > 0.05), while with different small letter superscripts mean significant different (*p* < 0.05). the letter of “a”, ”b” and ”c” were used to distinguish significance in the same row for each serological indices.

## Data Availability

All the RNA-seq datasets have been deposited in the NCBI Short Read database under the accession number PRJNA757552.

## Supplementary Materials

The following supporting information can be downloaded at: https://www.mdpi.com/article/10.3390/genes13010089/s1, Figure S1: Cluster Analysis of DEGs from hypothalamic (a) and ovarian (b) samples in the group of FM_1-vs-NM_1, hypothalamic (c) and ovarian (d) samples in the group of FM_2-vs-NM_2, hypothalamic (e) and ovarian (f) samples in the group of FM_1-vs- FM_2, hypothalamic (g) and ovarian (h) samples in the group of NM_1-vs- NM_2. Table S1: Summary statistics for sequence quality of 24 samples. Table S2: Partial representative GO terms and KEGG pathways classified by specific function descriptions in each group. Table S3: Partial representative DEGe descriptions in each group; Additional file S1: The DEGs in hypothalamus from the group of FM_1-vs-NM_1; Additional file S2: The GO and KEGG analysis of DEGs in hypothalamus from the group of FM_1-vs-NM_1; Additional file S3: The DEGs of ovary in the group of FM_1-vs-NM_1; Additional file S4: The GO and KEGG analysis of DEGs in ovary from the group of FM_1-vs-NM_1; Additional file S5: The DEGs of hypothalamus in the group of FM_2-vs-NM_2; Additional file S6: The GO and KEGG analysis of DEGs in hypothalamus from the group of FM_2-vs-NM_2; Additional file S7: The DEGs of ovary in the group of FM_2-vs-NM_2; Additional file S8 The GO and KEGG analysis of DEGs in ovary from the group of FM_2-vs-NM_2; Additional file S9: The DEGs of hypothalamus in the group of FM_1-vs-FM_2. The DEGs of hypothalamus in the group of NM_1-vs-NM_2; Additional file S10: The GO and KEGG analysis of DEGs in hypothalamus from the group of FM_1-vs-FM_2; Additional file S11: The DEGs of ovary in the group of FM_1-vs-FM_2 (The 537 unique DEGs was colored by yellow). The DEGs of ovary in the group of NM_1-vs-NM_2 (The 524 unique DEGs was colored by yellow); Additional file S12: The GO and KEGG analysis of unique ovarian DEGs in from the group of FM_1-vs-FM_2. The GO and KEGG analysis of unique ovarian DEGs in from the group of NM_1-vs-NM_2.
