# Supplementary material for: Understanding Transcriptomic and Serological Differences between Forced Molting and Natural Molting in Laying Hens
Source: Genes (Basel). 2021 Dec 29;13(1):89. doi: 10.3390/genes13010089 (PMC8774386; doi:10.3390/genes13010089)
Supplement: Supplementary file 1 [file genes-13-00089-s001.zip › Supplementary Materials.pdf]

Supplementary materials

Figure S1 Cluster Analysis of DEGs from hypothalamic (a) and ovarian (b) samples in the group of FM_1-vs-NM_1, hypothalamic (c) and ovarian (d) samples in the group of FM_2-vs-NM_2, hypothalamic (e) and ovarian (f) samples in the group of FM_1-vs- FM_2, hypothalamic (g) and ovarian (h) samples in the group of NM_1-vs- NM_2. Colored bars indicate the expression levels. Each column represents one sample and each row represents one differential DEG. Color scale from red to green in the heatmap represents the normalized FPKM using Row Z-score, Values higher than the row mean are represented in red, and values lower than the row mean are represented in blue. Red and blue color intensity indicate gene upregulation and downregulation, respectively.

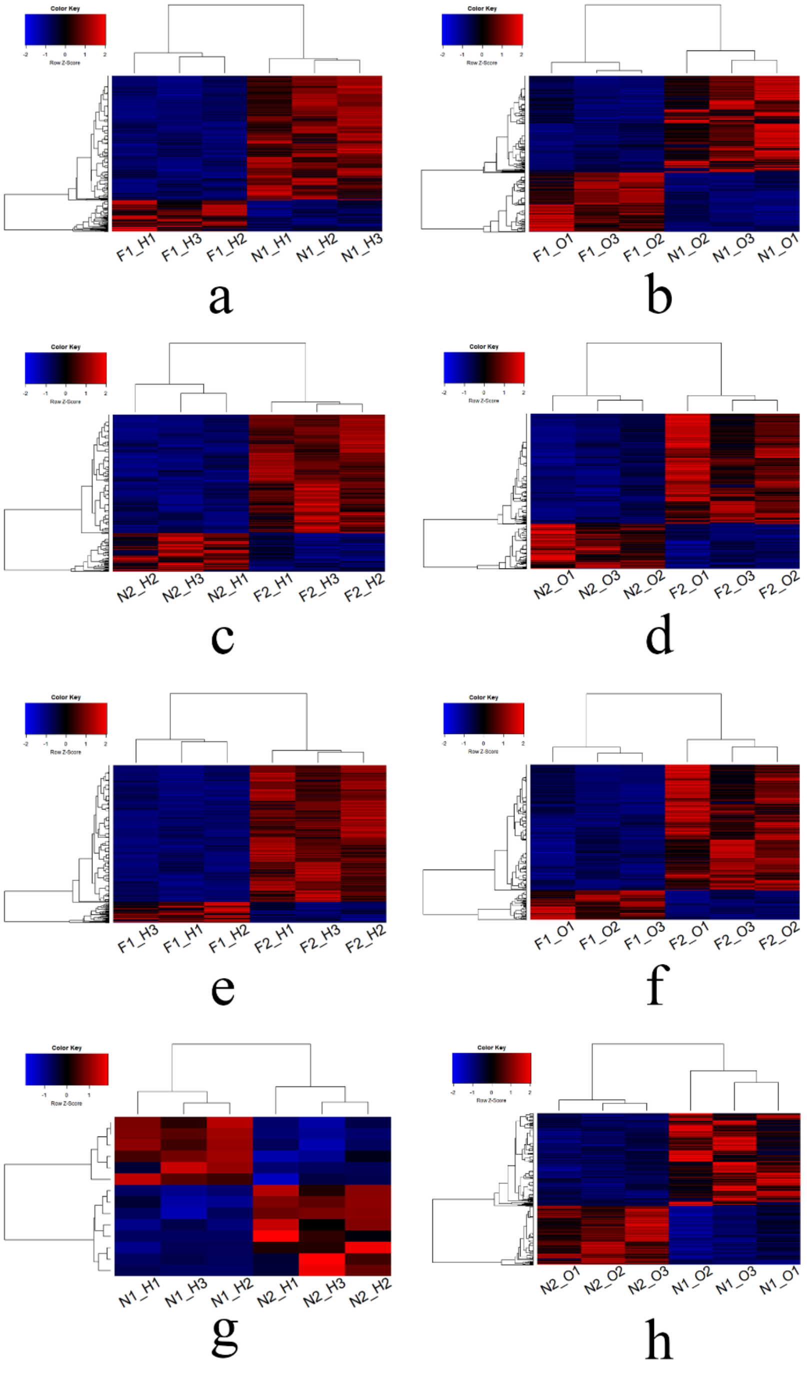

Table S1 Summary statistics for sequence quality of 24 samples

Samples	Raw Reads (106)	Raw bases (106)	Clean Reads (106)	Clean bases (106)	Q20 (%)	Q30 (%)	GC content (%)
FM_1-1-o	40.87	6130.03	37.91	5686.19	97.68	94.24	50.54
FM_1-1-h	41.72	6258.21	38.75	5812.03	97.55	93.96	49.25
FM_1-2-o	42.48	6372.62	39.22	5882.57	97.16	92.55	49.39
FM_1-2-h	44.55	6682.44	41.36	6203.45	97.62	94.01	49.33
FM_1-3-o	38.63	5794.97	35.95	5392.27	97.69	94.21	49.53
FM_1-3-h	41.55	6232.85	38.54	5781.74	97.41	93.59	49.48
FM_2-1-o	53.25	7987.22	49.03	7353.82	97.72	93.96	47.86
FM_2-1-h	47.07	7060.75	43.73	6558.82	97.65	93.84	47.48
FM_2-2-o	49.82	7472.76	46.11	6916.56	97.72	93.94	47.58
FM_2-2-h	44.44	6666.1	41.34	6200.93	97.41	93.38	47.39
FM_2-3-o	50.34	7551.63	46.18	6926.7	97.35	93.3	48.14
FM_2-3-h	45.91	6886.34	42.7	6404.44	97.51	93.58	47.28
NM_1-1-o	49.43	7414.66	48.8	7308.53	97.92	94.15	51.33
NM_1-1-h	39.29	5894.87	38.88	5826.02	97.55	93.42	48.35
NM_1-2-o	46.45	6967.51	45.94	6882.3	97.58	93.54	49.96
NM_1-2-h	44.01	6601.79	43.58	6528.7	97.99	94.1	48.26
NM_1-3-o	43.67	6551.57	43.08	6452.51	97.75	93.7	50.97
NM_1-3-h	47.16	7073.53	46.66	6991.2	97.83	93.69	48.25
NM_2-1-o	42.26	6338.28	41.81	6263.83	97.69	93.82	50.53
NM_2-1-h	40.31	6046.56	39.89	5976.42	98.01	94.18	48.27
NM_2-2-o	42.39	6359.14	41.99	6291.7	97.69	93.19	49.57
NM_2-2-h	46.1	6914.92	45.6	6831.96	97.92	93.94	47.63
NM_2-3-o	41.5	6225.17	41.06	6151.98	97.94	94.04	49.56
NM_2-3-h	45.37	6805.39	44.93	6731.35	98.03	94.23	48.05

Note: FM_i-j-o (i=1,2; j=1,2,3) represent ovary samples from 3 biological replicas in 2 periods of forced molting; FM_i-j-h (i=1,2; j=1,2,3) represent hypothalamus samples from 3 biological replicas in 2 periods of forced molting;

NM_i-j-o (i=1,2; j=1,2,3) represent ovary samples from 3 biological replicas in 2 periods of natural molting; NM_i-j-h (i=1,2; j=1,2,3) represent hypothalamus samples from 3 biological replicas in 2 periods of natural molting;

Table S2 Partial representative GO terms and KEGG pathways classified by specific function descriptions in each group.

Groups	Function	Pvalue	GO_Terms
H(FM_1-vs-NM_1)	immune	0.003	GO:0019885~antigen processing and presentation of endogenous peptide antigen via MHC class I
H(FM_1-vs-NM_1)	immune	0.007	GO:0019731~antibacterial humoral response
H(FM_1-vs-NM_1)	immune	0.008	GO:0045087~innate immune response
H(FM_1-vs-NM_1)	immune	0.039	GO:0050766~positive regulation of phagocytosis
H(FM_1-vs-NM_1)	energy metabolism	0.044	GO:0015986~ATP synthesis coupled proton transport
H(FM_1-vs-NM_1)	energy metabolism	0.045	GO:0032812~positive regulation of epinephrine secretion
H(FM_1-vs-NM_1)	energy metabolism	0.000	GO:0022625~cytosolic large ribosomal subunit
H(FM_1-vs-NM_1)	energy metabolism	0.000	GO:0022627~cytosolic small ribosomal subunit
H(FM_1-vs-NM_1)	energy metabolism	0.000	GO:0003735~structural constituent of ribosome
H(FM_1-vs-NM_1)	energy metabolism	0.044	GO:0000027~ribosomal large subunit assembly
H(FM_1-vs-NM_1)	energy metabolism	0.050	GO:0004129~cytochrome-c oxidase activity
H(FM_1-vs-NM_1)	energy metabolism	0.002	gga04260:Cardiac muscle contraction
O(FM_1-vs-NM_1)	immune	0.017	GO:0006955~immune response
O(FM_1-vs-NM_1)	immune	0.034	GO:0010745~negative regulation of macrophage derived foam cell differentiation
O(FM_1-vs-NM_1)	immune	0.034	GO:0002726~positive regulation of T cell cytokine production
O(FM_1-vs-NM_1)	immune	0.049	GO:0006898~receptor-mediated endocytosis
O(FM_1-vs-NM_1)	development	0.023	GO:0021772~olfactory bulb development
O(FM_1-vs-NM_1)	development	0.034	GO:0043009~chordate embryonic development
O(FM_1-vs-NM_1)	development	0.039	GO:0001889~liver development
O(FM_1-vs-NM_1)	development	0.048	GO:0030325~adrenal gland development
O(FM_1-vs-NM_1)	aging	0.017	GO:0007155~cell adhesion
O(FM_1-vs-NM_1)	aging	0.001	GO:0008137~NADH dehydrogenase (ubiquinone) activity
O(FM_1-vs-NM_1)	aging	0.049	gga02010:ABC transporters
O(FM_1-vs-NM_1)	aging	0.039	gga04150:mTOR signaling pathway
O(FM_1-vs-NM_1)	aging	0.000	gga04510:Focal adhesion
O(FM_1-vs-NM_1)	aging	0.000	gga03010:Ribosome
H(FM_2-vs-NM_2)	development	0.000	GO:0007275~multicellular organism development
H(FM_2-vs-NM_2)	development	0.024	GO:0048732~gland development
H(FM_2-vs-NM_2)	development	0.006	GO:0048704~embryonic skeletal system morphogenesis
H(FM_2-vs-NM_2)	immune	0.000	GO:0006954~inflammatory response
H(FM_2-vs-NM_2)	immune	0.040	GO:0050852~T cell receptor signaling pathway
H(FM_2-vs-NM_2)	immune	0.044	GO:0006955~immune response
O(FM_2-vs-NM_2)	immune	0.000	GO:0007155~cell adhesion
O(FM_2-vs-NM_2)	immune	0.003	GO:0050727~regulation of inflammatory response
O(FM_2-vs-NM_2)	immune	0.027	GO:0016337~single organismal cell-cell adhesion
O(FM_2-vs-NM_2)	immune	0.042	GO:0034446~substrate adhesion-dependent cell spreading
O(FM_2-vs-NM_2)	immune	0.042	GO:0006955~immune response
O(FM_2-vs-NM_2)	immune	0.039	gga05132:Salmonella infection
O(FM_2-vs-NM_2)	development	0.002	GO:0030324~lung development

O(FM_2-vs-NM_2)	development	0.002	GO:0001889~liver development
O(FM_2-vs-NM_2)	development	0.016	GO:0001822~kidney development
O(FM_2-vs-NM_2)	development	0.015	GO:0002076~osteoblast development
O(FM_2-vs-NM_2)	development	0.032	GO:0072358~cardiovascular system development
O(FM_2-vs-NM_2)	aging	0.042	gga00750:Vitamin B6 metabolism
O(FM_2-vs-NM_2)	aging	0.011	GO:0048754~branching morphogenesis of an epithelial tube
O(FM_2-vs-NM_2)	aging	0.032	GO:0010595~positive regulation of endothelial cell migration
O(FM_2-vs-NM_2)	reproduction	0.000	GO:0005509~calcium ion binding
O(FM_2-vs-NM_2)	reproduction	0.001	gga04020:Calcium signaling pathway
O(FM_2-vs-NM_2)	reproduction	0.001	gga04912:GnRH signaling pathway
H(FM_1-vs-FM_2)	development	0.000	GO:0007275~multicellular organism development
H(FM_1-vs-FM_2)	development	0.011	GO:0048704~embryonic skeletal system morphogenesis
H(FM_1-vs-FM_2)	development	0.016	GO:0051216~cartilage development
H(FM_1-vs-FM_2)	development	0.024	GO:0030855~epithelial cell differentiation
H(FM_1-vs-FM_2)	development	0.031	GO:0048536~spleen development
H(FM_1-vs-FM_2)	anti-aging	0.000	gga00190:Oxidative phosphorylation
H(FM_1-vs-FM_2)	development	0.004	gga04350:TGF-beta signaling pathway
H(FM_1-vs-FM_2)	apoptosis	0.038	GO:0043524~negative regulation of neuron apoptotic process
H(FM_1-vs-FM_2)	Hunger stress	0.039	GO:0032099~negative regulation of appetite
H(FM_1-vs-FM_2)	energy metabolism	0.028	gga01100:Metabolic pathways
H(FM_1-vs-FM_2)	energy metabolism	0.000	gga03010:Ribosome
H(FM_1-vs-FM_2)	energy metabolism	0.006	GO:0008137~NADH dehydrogenase (ubiquinone) activity
H(FM_1-vs-FM_2)	energy metabolism	0.048	GO:0022627~cytosolic small ribosomal subunit
U_O(FM_1-vs-FM_2)	energy metabolism	0.000	gga03010:Ribosome
U_O(FM_1-vs-FM_2)	anti-aging	0.000	gga00190:Oxidative phosphorylation
U_O(FM_1-vs-FM_2)	development	0.023	gga04350:TGF-beta signaling pathway
U_O(FM_1-vs-FM_2)	energy metabolism	0.000	GO:0003735~structural constituent of ribosome
U_O(FM_1-vs-FM_2)	energy metabolism	0.015	GO:0022627~cytosolic small ribosomal subunit
U_O(FM_1-vs-FM_2)	development	0.040	GO:0044822~poly(A) RNA binding
U_O(NM_1-vs-NM_2)	immune	0.008	GO:0007155~cell adhesion
U_O(NM_1-vs-NM_2)	development	0.029	GO:0048566~embryonic digestive tract development
U_O(NM_1-vs-NM_2)	development	0.034	GO:0060173~limb development
U_O(NM_1-vs-NM_2)	development	0.040	GO:0048468~cell development
U_O(NM_1-vs-NM_2)	development	0.044	GO:0003166~bundle of His development
U_O(NM_1-vs-NM_2)	development	0.044	GO:0060959~cardiac neuron development
U_O(NM_1-vs-NM_2)	health	0.875	gga00260:Glycine, serine and threonine metabolism
U_O(NM_1-vs-NM_2)	health	0.656	gga00220:Arginine biosynthesis

Note: H(FM_1-vs-NM_1) means hypothalamus in the group of FM_1-vs-NM_1, O(FM_1-vs-NM_1) means ovary in the group of FM_1-vs-NM_1, U_O(FM_1-vs-FM_2) means unique ovary in the group of FM_1-vs-FM_2, U_O(NM_1-vs-NM_2) means unique ovary in the group of NM_1-vs-NM_2, and the other groups fared similarly.

Table S3 Partial representative DEGe descriptions in each group.

Genes	GO Terms/KEGG Pathways	Groups	Log2FC	Over expression	Discription
MX1	GO:0045087~innate immune response	H(FM_1-vs-NM_1)	-1.704	FM_1	MX dynamin like GTPase 1
PTX3	GO:0045088~innate immune response	H(FM_1-vs-NM_1)	-1.002	FM_1	pentraxin 3
SMAD3	GO:0006955~immune response	O(FM_1-vs-NM_1)	1.865	NM_1	SMAD family member 3
CCL5	GO:0006955~immune response	O(FM_1-vs-NM_1)	-1.540	FM_1	C-C motif chemokine ligand 5
ABCA5	GO:0010745~negative regulation of macrophage derived foam cell differentiation	O(FM_1-vs-NM_1)	-1.758	FM_1	ATP binding cassette subfamily A member 5
ADIPOQ	GO:0010745~negative regulation of macrophage derived foam cell differentiation	O(FM_1-vs-NM_1)	2.059	NM_1	adiponectin, C1Q and collagen domain containing
ITGAV	GO:0010745~negative regulation of macrophage derived foam cell differentiation	O(FM_1-vs-NM_1)	-2.135	FM_1	integrin subunit alpha V
PPARG	GO:0010745~negative regulation of macrophage derived foam cell differentiation	O(FM_1-vs-NM_1)	-1.310	FM_1	peroxisome proliferator activated receptor gamma
FOXD3	GO:0043009~chordate embryonic development	O(FM_1-vs-NM_1)	2.361	NM_1	forkhead box D3
WT1	GO:0043009~chordate embryonic development	O(FM_1-vs-NM_1)	-1.237	FM_1	WT1 transcription factor
BRCA1	GO:0043009~chordate embryonic development	O(FM_1-vs-NM_1)	-1.368	FM_1	BRCA1 DNA repair associated
BRCA2	GO:0043009~chordate embryonic development	O(FM_1-vs-NM_1)	-1.231	FM_1	BRCA2 DNA repair associated
HOXA3	GO:0007275~multicellular organism development	H(FM_2-vs-NM_2)	-2.981	FM_2	homeobox A3
HOXB3	GO:0007275~multicellular organism development	H(FM_2-vs-NM_2)	-2.531	FM_2	homeobox B3
HOXD4	GO:0007275~multicellular organism development	H(FM_2-vs-NM_2)	-4.053	FM_2	homeobox D4
HOXA4	GO:0007275~multicellular organism development	H(FM_2-vs-NM_2)	-3.199	FM_2	homeobox A4
HOXB5	GO:0007275~multicellular organism development	H(FM_2-vs-NM_2)	-4.464	FM_2	homeobox B5
MAP3K2	gga04912:GnRH signaling pathway	O(FM_2-vs-NM_2)	1.166	NM_2	mitogen-activated protein kinase kinase kinase 2
SLITRK5	GO:0072358~cardiovascular system development	O(FM_2-vs-NM_2)	1.350	NM_2	SLIT and NTRK like family member 5

PDK4	NA	H(NM_1-vs-NM_2)	1.127	NM_2	pyruvate dehydrogenase kinase 4
HTR3A	NA	H(NM_1-vs-NM_2)	1.145	NM_2	5-hydroxytryptamine receptor 3A
ZBTB32	NA	H(NM_1-vs-NM_2)	1.202	NM_2	zinc finger and BTB domain containing 32
CD69L	NA	H(NM_1-vs-NM_2)	-1.140	NM_1	CD69 molecule like
NDUFB6	gga00190:Oxidative phosphorylation	H(FM_1-vs-FM_2)	-1.147	FM_1	NADH:ubiquinone oxidoreductase subunit B6
NDUFS5	gga00190:Oxidative phosphorylation	H(FM_1-vs-FM_2)	-1.659	FM_1	NADH:ubiquinone oxidoreductase subunit S5
NDUFB6	gga00190:Oxidative phosphorylation	O(FM_1-vs-FM_2)	-1.021	FM_1	NADH:ubiquinone oxidoreductase subunit B6
NDUFS5	gga00190:Oxidative phosphorylation	O(FM_1-vs-FM_2)	-1.356	FM_1	NADH:ubiquinone oxidoreductase subunit S5

Note: H(FM_1-vs-NM_1) means hypothalamus in the group of FM_1-vs-NM_1, O(FM_1-vs-NM_1) means ovary in the group of FM_1-vs-NM_1, and the other groups fared similarly.

Additional file (S1-S12)

Additional file S1 The DEGs in hypothalamus from the group of FM_1-vs-NM_1.

Additional file S2 The GO and KEGG analysis of DEGs in hypothalamus from the group of FM_1-vs-NM_1.

Additional file S3 The DEGs of ovary in the group of FM_1-vs-NM_1.

Additional file S4 The GO and KEGG analysis of DEGs in ovary from the group of FM_1-vs-NM_1.

Additional file S5 The DEGs of hypothalamus in the group of FM_2-vs-NM_2.

Additional file S6 The GO and KEGG analysis of DEGs in hypothalamus from the group of FM_2-vs-NM_2.

Additional file S7 The DEGs of ovary in the group of FM_2-vs-NM_2.

Additional file S8 The GO and KEGG analysis of DEGs in ovary from the group of FM_2-vs-NM_2.

Additional file S9 The DEGs of hypothalamus in the group of FM_1-vs-FM_2. The DEGs of hypothalamus in the group of NM_1-vs-NM_2.

Additional file S10 The GO and KEGG analysis of DEGs in hypothalamus from the group of FM_1-vs-FM_2.

Additional file S11 The DEGs of ovary in the group of FM_1-vs-FM_2 (The 537 unique DEGs was colored by yellow). The DEGs of ovary in the group of NM_1-vs-NM_2 (The 524 unique DEGs was colored by yellow).

Additional file S12 The GO and KEGG analysis of unique ovarian DEGs in from the group of FM_1-vs-FM_2. The GO and KEGG analysis of unique ovarian DEGs in from the group of NM_1-vs-NM_2.
